# *N*′-[(*E*)-(5-Bromo-2-hydroxy­phen­yl)(phen­yl)methyl­ene]benzohydrazide

**DOI:** 10.1107/S1600536809002918

**Published:** 2009-02-28

**Authors:** Chang-Zheng Zheng, Chang-You Ji, Xiu-Li Chang

**Affiliations:** a College of Environment and Chemical Engineering, Xi’an Polytechnic University, 710048 Xi’an, Shaanxi, People’s Republic of China; b Department of Material Science and Chemical Engineering, Sichuan University of Science and Engineering, 643000 Zigong, Sichuan, People’s Republic of China

## Abstract

In the title compound, C_20_H_15_BrN_2_O_2_, the C=N double bond displays a *trans* configuration. The crystal structure features an intra­molecular O—H⋯N hydrogen bond.

## Related literature

For literature on similar Schiff bases, see: Carcelli *et al.* (1995 ▶); Salem (1998 ▶); Singh *et al.* (1982 ▶).
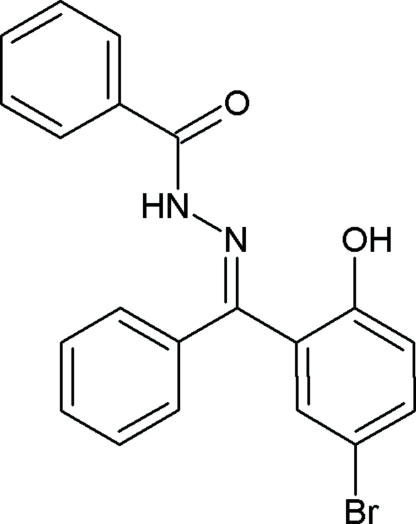


## Experimental

### 

#### Crystal data

C_20_H_15_BrN_2_O_2_
*M* *_r_* = 395.25
Monoclinic, 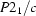

*a* = 17.505 (5) Å
*b* = 13.761 (4) Å
*c* = 7.219 (2) Å
β = 94.546 (6)°
*V* = 1733.4 (9) Å^3^
*Z* = 4
Mo *K*α radiation
μ = 2.39 mm^−1^
*T* = 298 (2) K
0.12 × 0.10 × 0.06 mm

#### Data collection

Bruker SMART diffractometer
Absorption correction: multi-scan (*SADABS*; Sheldrick, 1996 ▶) *T*_min_ = 0.763, *T*_max_ = 0.870
9019 measured reflections
3078 independent reflections
1722 reflections with *I* > 2σ(*I*)
*R*_int_ = 0.059

#### Refinement

*R*[*F* ^2^ > 2σ(*F* ^2^)] = 0.044
*wR*(*F* ^2^) = 0.101
*S* = 1.00
3078 reflections
197 parameters
H-atom parameters constrained
Δρ_max_ = 0.31 e Å^−3^
Δρ_min_ = −0.28 e Å^−3^

### 

Data collection: *SMART* (Bruker, 1996 ▶); cell refinement: *SAINT* (Bruker, 1996 ▶); data reduction: *SAINT*; program(s) used to solve structure: *SHELXS97* (Sheldrick, 2008 ▶); program(s) used to refine structure: *SHELXL97* (Sheldrick, 2008 ▶); molecular graphics: *SHELXTL* (Sheldrick, 2008 ▶); software used to prepare material for publication: *SHELXTL*.

## Supplementary Material

Crystal structure: contains datablocks I, global. DOI: 10.1107/S1600536809002918/ng2531sup1.cif

Structure factors: contains datablocks I. DOI: 10.1107/S1600536809002918/ng2531Isup2.hkl

Additional supplementary materials:  crystallographic information; 3D view; checkCIF report

## Figures and Tables

**Table 1:** Hydrogen-bond geometry (Å, °)

*D*—H⋯*A*	*D*—H	H⋯*A*	*D*⋯*A*	*D*—H⋯*A*
O1—H1⋯N1	0.82	1.85	2.562 (4)	145

**Fig. 1.**
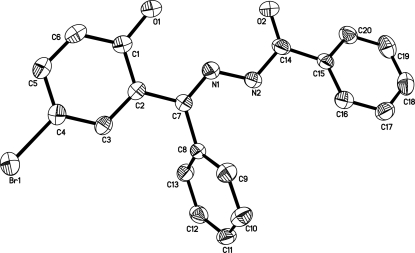
The molecular structure of compound (I), showing the atom-numbering scheme. Displacement ellipsoids are drawn at the 30% probability level.

**Fig. 2.**
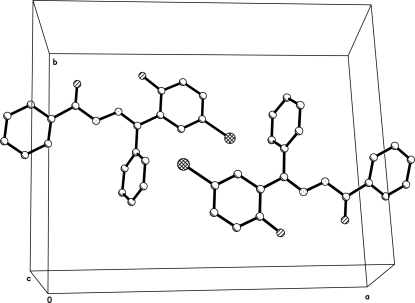
The crystal packing of (I), viewed along the c axis. Dashed lines show intra-and intermolecular hydrogen bonds.

## supplementary crystallographic information

### Comment

The chemistry of aroylhydrazones continues to attract much attention due to their coordination ability to metal ions (Singh *et al.*, 1982; Salem, 1998) and their biological activity (Singh *et al.*, 1982; Carcelli *et al.*, 1995).As an extension of work on the structural characterization of aroylhydrazone derivatives,the title compound, (I),was synthesized and its crystal structure is reported here.

The title molecule displays a *trans* conformation with respect to the C8=N1 double bond (Fig. 1). The crystal structure is stabilized by intramolecular O—H···N and intermolecular N—H···O hydrogen bonds (Table 1. and Fig. 2).

### Experimental

benzoylhydrazine (0.02 mol,2.72 g) was dissolved in anhydrous ethanol (50 ml), and (5-bromo-2-hydroxyphenyl)(phenyl)methanone (0.02 mol, 5.54 g) was added. The reaction mixture was refluxed for 6 h with stirring, then the resulting precipitate was collected by filtration, washed several times with ethanol and dried *in vacuo* (yield 85%). The compound (2.0 mmol,0.79 g) was dissolved in dimethylformamide (30 ml) and kept at room temperature for 30 d to obtain yellow single crystals suitable for X-ray diffraction.

### Refinement

All H atoms were positioned geometrically and treated as riding on their parent atoms,with C—H(aromatic) = 0.93 Å, O—H = 0.82 Å, and N—H = 0.86 Å and with *U*_iso_(H) = 1.2*U*_eq_(C_aromatic_,*N*).

### Crystal data

**Table:**

C_20_H_15_BrN_2_O_2_	*F*(000) = 800
*M**_r_* = 395.25	*D*_x_ = 1.515 Mg m^−^^3^
Monoclinic, *P*2_1_/*c*	Mo *K*α radiation, λ = 0.71073 Å
Hall symbol: -P 2ybc	Cell parameters from 1125 reflections
*a* = 17.505 (5) Å	θ = 2.3–17.9°
*b* = 13.761 (4) Å	µ = 2.39 mm^−^^1^
*c* = 7.219 (2) Å	*T* = 298 K
β = 94.546 (6)°	Block, yellow
*V* = 1733.4 (9) Å^3^	0.12 × 0.10 × 0.06 mm
*Z* = 4	

### Data collection

**Table:**

Bruker SMART diffractometer	3078 independent reflections
Radiation source: fine-focus sealed tube	1722 reflections with *I* > 2σ(*I*)
graphite	*R*_int_ = 0.059
φ and ω scans	θ_max_ = 25.1°, θ_min_ = 1.9°
Absorption correction: multi-scan (*SADABS*; Sheldrick, 1996)	*h* = −19→20
*T*_min_ = 0.763, *T*_max_ = 0.870	*k* = −13→16
9019 measured reflections	*l* = −8→7

### Refinement

**Table:**

Refinement on *F*^2^	Primary atom site location: structure-invariant direct methods
Least-squares matrix: full	Secondary atom site location: difference Fourier map
*R*[*F*^2^ > 2σ(*F*^2^)] = 0.044	Hydrogen site location: inferred from neighbouring sites
*wR*(*F*^2^) = 0.101	H-atom parameters constrained
*S* = 1.00	*w* = 1/[σ^2^(*F*_o_^2^) + (0.038*P*)^2^ + 0.0901*P*] where *P* = (*F*_o_^2^ + 2*F*_c_^2^)/3
3078 reflections	(Δ/σ)_max_ < 0.001
197 parameters	Δρ_max_ = 0.31 e Å^−^^3^
0 restraints	Δρ_min_ = −0.28 e Å^−^^3^

### Special details

**Table:**

Geometry. All e.s.d.'s (except the e.s.d. in the dihedral angle between two l.s. planes) are estimated using the full covariance matrix. The cell e.s.d.'s are taken into account individually in the estimation of e.s.d.'s in distances, angles and torsion angles; correlations between e.s.d.'s in cell parameters are only used when they are defined by crystal symmetry. An approximate (isotropic) treatment of cell e.s.d.'s is used for estimating e.s.d.'s involving l.s. planes.
Refinement. Refinement of *F*^2^ against ALL reflections. The weighted *R*-factor *wR* and goodness of fit *S* are based on *F*^2^, conventional *R*-factors *R* are based on *F*, with *F* set to zero for negative *F*^2^. The threshold expression of *F*^2^ > σ(*F*^2^) is used only for calculating *R*-factors(gt) *etc*. and is not relevant to the choice of reflections for refinement. *R*-factors based on *F*^2^ are statistically about twice as large as those based on *F*, and *R*- factors based on ALL data will be even larger.

### Fractional atomic coordinates and isotropic or equivalent isotropic displacement parameters (Å^2^)

**Table:**

	*x*	*y*	*z*	*U*_iso_*/*U*_eq_	
Br1	0.42053 (3)	0.11003 (4)	0.37885 (7)	0.0690 (2)	
O1	0.69581 (16)	0.35312 (19)	0.2430 (4)	0.0600 (8)	
H1	0.7343	0.3203	0.2327	0.090*	
O2	0.89683 (17)	0.2957 (2)	0.1320 (5)	0.0729 (9)	
N1	0.7724 (2)	0.1958 (2)	0.2165 (4)	0.0524 (9)	
N2	0.84133 (19)	0.1530 (2)	0.1895 (5)	0.0573 (9)	
H2	0.8456	0.0908	0.1935	0.069*	
C1	0.6363 (2)	0.2941 (3)	0.2723 (5)	0.0469 (10)	
C2	0.6425 (2)	0.1921 (3)	0.2769 (5)	0.0428 (10)	
C3	0.5771 (2)	0.1384 (3)	0.3098 (5)	0.0463 (10)	
H3	0.5799	0.0710	0.3158	0.056*	
C4	0.5090 (2)	0.1842 (3)	0.3332 (5)	0.0495 (11)	
C5	0.5032 (3)	0.2842 (3)	0.3297 (5)	0.0548 (11)	
H5	0.4571	0.3148	0.3481	0.066*	
C6	0.5670 (3)	0.3371 (3)	0.2983 (5)	0.0566 (12)	
H6	0.5635	0.4045	0.2944	0.068*	
C7	0.7149 (2)	0.1422 (3)	0.2494 (5)	0.0441 (10)	
C8	0.7210 (2)	0.0332 (3)	0.2583 (6)	0.0419 (10)	
C9	0.7594 (2)	−0.0103 (3)	0.4116 (6)	0.0553 (12)	
H9	0.7792	0.0275	0.5109	0.066*	
C10	0.7682 (3)	−0.1098 (3)	0.4170 (6)	0.0641 (12)	
H10	0.7951	−0.1388	0.5187	0.077*	
C11	0.7376 (3)	−0.1661 (3)	0.2735 (7)	0.0608 (12)	
H11	0.7434	−0.2332	0.2783	0.073*	
C12	0.6986 (2)	−0.1235 (3)	0.1229 (7)	0.0579 (12)	
H12	0.6768	−0.1620	0.0268	0.069*	
C13	0.6915 (2)	−0.0232 (3)	0.1130 (6)	0.0513 (11)	
H13	0.6667	0.0057	0.0083	0.062*	
C14	0.9034 (2)	0.2088 (3)	0.1563 (6)	0.0527 (11)	
C15	0.9765 (2)	0.1545 (3)	0.1514 (5)	0.0471 (10)	
C16	0.9902 (3)	0.0672 (3)	0.2383 (6)	0.0570 (12)	
H16	0.9524	0.0391	0.3043	0.068*	
C17	1.0594 (3)	0.0201 (3)	0.2294 (6)	0.0652 (13)	
H17	1.0681	−0.0391	0.2895	0.078*	
C18	1.1156 (3)	0.0609 (4)	0.1314 (7)	0.0701 (14)	
H18	1.1622	0.0293	0.1232	0.084*	
C19	1.1021 (3)	0.1485 (4)	0.0465 (7)	0.0726 (14)	
H19	1.1401	0.1766	−0.0188	0.087*	
C20	1.0337 (3)	0.1958 (3)	0.0553 (6)	0.0584 (12)	
H20	1.0256	0.2556	−0.0031	0.070*	

### Atomic displacement parameters (Å^2^)

**Table:**

	*U*^11^	*U*^22^	*U*^33^	*U*^12^	*U*^13^	*U*^23^
Br1	0.0514 (3)	0.0762 (4)	0.0801 (4)	0.0014 (3)	0.0099 (2)	0.0023 (3)
O1	0.065 (2)	0.0412 (17)	0.074 (2)	−0.0003 (15)	0.0062 (18)	−0.0016 (16)
O2	0.063 (2)	0.0396 (18)	0.115 (3)	−0.0032 (16)	0.0033 (18)	0.0054 (19)
N1	0.049 (2)	0.047 (2)	0.062 (2)	−0.0002 (19)	0.0078 (18)	−0.0026 (18)
N2	0.047 (2)	0.042 (2)	0.084 (3)	−0.0039 (18)	0.0108 (19)	−0.0040 (19)
C1	0.057 (3)	0.043 (3)	0.041 (2)	0.001 (2)	0.003 (2)	0.000 (2)
C2	0.050 (3)	0.045 (3)	0.034 (2)	0.007 (2)	0.0050 (19)	0.001 (2)
C3	0.049 (3)	0.049 (3)	0.041 (2)	0.007 (2)	−0.0006 (19)	0.003 (2)
C4	0.047 (3)	0.061 (3)	0.041 (2)	0.005 (2)	0.0044 (19)	0.001 (2)
C5	0.054 (3)	0.056 (3)	0.054 (3)	0.013 (2)	0.002 (2)	−0.005 (2)
C6	0.073 (3)	0.041 (3)	0.055 (3)	0.011 (3)	0.000 (2)	−0.001 (2)
C7	0.044 (3)	0.044 (3)	0.044 (2)	−0.002 (2)	0.0040 (19)	−0.001 (2)
C8	0.037 (2)	0.039 (3)	0.050 (3)	−0.0013 (19)	0.0071 (19)	0.002 (2)
C9	0.062 (3)	0.052 (3)	0.051 (3)	0.003 (2)	0.003 (2)	−0.003 (2)
C10	0.075 (3)	0.056 (3)	0.062 (3)	0.012 (3)	0.008 (2)	0.013 (3)
C11	0.063 (3)	0.040 (3)	0.083 (4)	0.005 (2)	0.024 (3)	0.006 (3)
C12	0.054 (3)	0.051 (3)	0.070 (3)	−0.007 (2)	0.012 (2)	−0.012 (3)
C13	0.051 (3)	0.048 (3)	0.054 (3)	0.005 (2)	0.002 (2)	−0.001 (2)
C14	0.054 (3)	0.044 (3)	0.060 (3)	−0.012 (2)	0.000 (2)	−0.003 (2)
C15	0.046 (3)	0.043 (3)	0.052 (3)	−0.008 (2)	−0.001 (2)	−0.003 (2)
C16	0.058 (3)	0.051 (3)	0.062 (3)	−0.004 (2)	0.005 (2)	−0.001 (3)
C17	0.074 (4)	0.049 (3)	0.070 (3)	0.005 (3)	−0.012 (3)	−0.001 (3)
C18	0.050 (3)	0.084 (4)	0.075 (4)	0.009 (3)	−0.003 (3)	−0.022 (3)
C19	0.051 (3)	0.101 (4)	0.067 (3)	−0.012 (3)	0.011 (2)	0.000 (3)
C20	0.057 (3)	0.060 (3)	0.058 (3)	−0.011 (3)	0.003 (2)	0.010 (2)

### Geometric parameters (Å, °)

**Table:**

Br1—C4	1.905 (4)	C9—C10	1.378 (5)
O1—C1	1.350 (4)	C9—H9	0.9300
O1—H1	0.8200	C10—C11	1.368 (5)
O2—C14	1.213 (4)	C10—H10	0.9300
N1—C7	1.284 (4)	C11—C12	1.369 (5)
N1—N2	1.370 (4)	C11—H11	0.9300
N2—C14	1.367 (5)	C12—C13	1.387 (5)
N2—H2	0.8600	C12—H12	0.9300
C1—C6	1.376 (5)	C13—H13	0.9300
C1—C2	1.409 (5)	C14—C15	1.484 (6)
C2—C3	1.397 (5)	C15—C16	1.369 (5)
C2—C7	1.469 (5)	C15—C20	1.384 (5)
C3—C4	1.370 (5)	C16—C17	1.380 (6)
C3—H3	0.9300	C16—H16	0.9300
C4—C5	1.380 (5)	C17—C18	1.377 (6)
C5—C6	1.368 (5)	C17—H17	0.9300
C5—H5	0.9300	C18—C19	1.364 (7)
C6—H6	0.9300	C18—H18	0.9300
C7—C8	1.506 (5)	C19—C20	1.370 (6)
C8—C13	1.373 (5)	C19—H19	0.9300
C8—C9	1.384 (5)	C20—H20	0.9300
			
C1—O1—H1	109.5	C11—C10—H10	119.8
C7—N1—N2	119.5 (3)	C9—C10—H10	119.8
C14—N2—N1	120.3 (4)	C10—C11—C12	120.0 (4)
C14—N2—H2	119.9	C10—C11—H11	120.0
N1—N2—H2	119.9	C12—C11—H11	120.0
O1—C1—C6	117.5 (4)	C11—C12—C13	120.2 (4)
O1—C1—C2	123.0 (4)	C11—C12—H12	119.9
C6—C1—C2	119.4 (4)	C13—C12—H12	119.9
C3—C2—C1	117.9 (4)	C8—C13—C12	119.8 (4)
C3—C2—C7	120.2 (4)	C8—C13—H13	120.1
C1—C2—C7	121.8 (4)	C12—C13—H13	120.1
C4—C3—C2	120.7 (4)	O2—C14—N2	120.8 (4)
C4—C3—H3	119.7	O2—C14—C15	124.4 (4)
C2—C3—H3	119.7	N2—C14—C15	114.8 (4)
C3—C4—C5	121.3 (4)	C16—C15—C20	118.8 (4)
C3—C4—Br1	120.2 (3)	C16—C15—C14	123.5 (4)
C5—C4—Br1	118.5 (3)	C20—C15—C14	117.7 (4)
C6—C5—C4	118.3 (4)	C15—C16—C17	121.0 (4)
C6—C5—H5	120.8	C15—C16—H16	119.5
C4—C5—H5	120.8	C17—C16—H16	119.5
C5—C6—C1	122.3 (4)	C18—C17—C16	119.9 (5)
C5—C6—H6	118.8	C18—C17—H17	120.1
C1—C6—H6	118.8	C16—C17—H17	120.1
N1—C7—C2	117.1 (4)	C19—C18—C17	119.1 (5)
N1—C7—C8	121.7 (4)	C19—C18—H18	120.4
C2—C7—C8	121.2 (3)	C17—C18—H18	120.4
C13—C8—C9	119.7 (4)	C18—C19—C20	121.4 (5)
C13—C8—C7	120.6 (4)	C18—C19—H19	119.3
C9—C8—C7	119.6 (4)	C20—C19—H19	119.3
C10—C9—C8	119.9 (4)	C19—C20—C15	119.9 (4)
C10—C9—H9	120.1	C19—C20—H20	120.1
C8—C9—H9	120.1	C15—C20—H20	120.1
C11—C10—C9	120.3 (4)		
			
C7—N1—N2—C14	−179.3 (4)	C2—C7—C8—C9	−106.2 (4)
O1—C1—C2—C3	179.5 (3)	C13—C8—C9—C10	0.5 (6)
C6—C1—C2—C3	−0.6 (5)	C7—C8—C9—C10	−177.1 (4)
O1—C1—C2—C7	−0.1 (6)	C8—C9—C10—C11	−1.6 (6)
C6—C1—C2—C7	179.8 (3)	C9—C10—C11—C12	0.6 (6)
C1—C2—C3—C4	1.2 (5)	C10—C11—C12—C13	1.6 (6)
C7—C2—C3—C4	−179.2 (3)	C9—C8—C13—C12	1.6 (6)
C2—C3—C4—C5	−1.6 (6)	C7—C8—C13—C12	179.1 (4)
C2—C3—C4—Br1	−179.8 (3)	C11—C12—C13—C8	−2.6 (6)
C3—C4—C5—C6	1.3 (6)	N1—N2—C14—O2	−7.3 (6)
Br1—C4—C5—C6	179.5 (3)	N1—N2—C14—C15	173.2 (3)
C4—C5—C6—C1	−0.7 (6)	O2—C14—C15—C16	156.7 (4)
O1—C1—C6—C5	−179.8 (3)	N2—C14—C15—C16	−23.8 (5)
C2—C1—C6—C5	0.3 (6)	O2—C14—C15—C20	−22.5 (6)
N2—N1—C7—C2	−179.9 (3)	N2—C14—C15—C20	157.0 (4)
N2—N1—C7—C8	−0.3 (5)	C20—C15—C16—C17	−0.7 (6)
C3—C2—C7—N1	178.8 (3)	C14—C15—C16—C17	−179.8 (4)
C1—C2—C7—N1	−1.6 (5)	C15—C16—C17—C18	−0.2 (6)
C3—C2—C7—C8	−0.8 (5)	C16—C17—C18—C19	0.9 (7)
C1—C2—C7—C8	178.8 (3)	C17—C18—C19—C20	−0.6 (7)
N1—C7—C8—C13	−103.3 (4)	C18—C19—C20—C15	−0.3 (7)
C2—C7—C8—C13	76.3 (5)	C16—C15—C20—C19	0.9 (6)
N1—C7—C8—C9	74.2 (5)	C14—C15—C20—C19	−179.9 (4)

### Hydrogen-bond geometry (Å, °)

**Table:**

*D*—H···*A*	*D*—H	H···*A*	*D*···*A*	*D*—H···*A*
O1—H1···N1	0.82	1.85	2.562 (4)	145
